# Pathogenic role of different phenotypes of immune cells in airway allergic diseases: a study based on Mendelian randomization

**DOI:** 10.3389/fimmu.2024.1349470

**Published:** 2024-05-15

**Authors:** Zhihan Xu, Ren Li, Leigang Wang, Yisha Wu, Yuhe Tian, Yilin Su, Yuqiang Ma, Ruiying Li, Yao Wei, Chen Zhang, Shikai Han, Siyu Duan, Haiyi Peng, Jinmei Xue

**Affiliations:** ^1^ Department of Otolaryngology, Head and Neck Surgery, Second Hospital, Shanxi Medical University, Taiyuan, Shanxi, China; ^2^ Shanxi Key Laboratory of Rapid Diagnosis and Precision Treatment of Airway Allergic Diseases, Head & Neck Surgery, Second Hospital, Shanxi Medical University, Taiyuan, Shanxi, China; ^3^ Shanxi Airway Inflammatory Diseases Neuroimmunity Laboratory, Head & Neck Surgery, Second Hospital, Shanxi Medical University, Taiyuan, Shanxi, China; ^4^ Shanxi Medical University, Taiyuan, Shanxi, China; ^5^ Department of Environmental Health, School of Public Health, Shanxi Medical University, Taiyuan, Shanxi, China

**Keywords:** airway allergic disease, immunophenotype, immune cell, MR analysis, causal inference

## Abstract

**Background:**

Airway allergic disease (AAD) is a class of autoimmune diseases with predominantly Th2-type inflammation, mainly including allergic rhinitis (AR), allergic asthma (AS), and chronic sinusitis (CRS). There are very complex regulatory mechanisms between immune cells and AAD; however, previous reports found that the functions of the same immune cells in AAD are not identical.

**Objective:**

The aim of this study was to explore the causal relationship between different phenotypic immune cells and their association with AAD.

**Method:**

Utilizing the publicly available Genome-Wide Association Studies (GWAS) database, this study conducted a bidirectional Mendelian randomization (MR) to assess the causal relationship between immune cells of 731 different immunophenotypes and AAD. The primary assessment methods included inverse variance weighting, weighted median, and MR Egger. Additionally, sensitivity analyses such as MR-PRESSO, leave-one-out, and scatter plots were employed to eliminate the interference of heterogeneity and pleiotropy, ensuring the stability of the causal inference.

**Result:**

A total of 38 immune cells with different immunophenotypes were found to be positively and causally associated with AR, of which 26 were protective factors and 12 were risk factors. Positive associations were found between 33 immune cells and AS, of which 14 were protective factors and 19 were risk factors, as well as between 39 immune cells and CRS, of which 22 were protective factors and 17 were risk factors. Finally, the results of all relevant immune cells for the three diseases were taken and intersected, and it was found that CD3 on CD39+-activated Treg (IVW^AR^ = 0.001, IVW^CRS^ = 0.043, IVW^AS^ = 0.027) may be the key immune cell that inhibits the development of AAD (OR^AR^ = 0.940, OR^AS^ = 0.967, OR^CRS^ = 0.976).

**Conclusion:**

This study reveals that different immune phenotypes of immune cells are closely related to AAD at the genetic level, which provides a theoretical basis for future clinical studies.

## 1 Introduction

Airway allergic disease (AAD) is a class of allergic disease types characterized by infiltration of T helper 2 (Th2) cells; this mainly includes allergic rhinitis (AR), allergic asthma (AS), and chronic sinusitis (CRS) (especially CRS with nasal polyps) (1, 2). Th1/Th2 immune imbalance is an important factor leading to AAD. Th1 mainly mediates cellular immunity and secretes cytokines such as IFN-γ, IL-2, and so forth. IFN-γ inhibits the differentiation of Th2 cells as well as the synthesis of IgE by B cells and attenuate allergic reactions; Th2 mainly mediates humoral immunity and secretes cytokines such as IL-4/5. IL-4 further induces the differentiation of Th2 cells, which leads to the release of Th2-associated cytokines (IL-4/5/13), inhibits the proliferation of Th1 cells, and enhances the degranulation of mast cells, thereby exacerbating allergic reactions. When the body is exposed to allergens, antigen presenting cells secrete IL-10 to activate downstream CD4+T cells (Th0 cell) towards Th2 differentiation (3), Disruption of the Th1/Th2 balance is highly likely to result in allergic diseases.

The development of AAD involves multiple immune cell regulation, and eosinophils (EOS) are common tissue infiltrating cells in AAD; when the body is sensitized, EOS not only converges toward the inflammatory response and releases heterophilic basic proteins to kill bacteria or helminths but also causes damage to surrounding tissues. EOS can also release a variety of inflammatory mediators that exacerbate inflammation (IL-3, IL-5, Colony stimulating factors) (4); B cell is also one of the key cells in the development of AAD. After activation of B cell, it produces IgE, which binds to and activates mast cell degranulation, releasing active mediators such as leukotrienes and histamine, and triggering an Type I–hypersensitivity reaction. Regulatory T cells (Treg) are crucial immunoregulatory cells that carry out immunosuppressive functions, which are crucial in maintaining the body’s immune balance and reducing Th2-type inflammation. The expression level and function of Treg and their key transcription factor Foxp3 are significantly reduced under the stimulation of multiple pathogenic factors (5), Accordingly, the immunosuppressive function of Treg is diminished, which is one of the pathogenic mechanisms of many allergic diseases, including AAD.

Different subsets of the same immune cells have different functions, such as T cells, which have the potential to differentiate into CD4+ T cells and CD8+ T cells, and CD4+ T cells can further differentiate into Th1, Th2, Th17, Treg, and other T-helper cell subsets. The mainstream view is that Th2 and Th17 are associated with an increased risk of AAD, while Treg has immunosuppressive effects; in addition, it has been found that the same immune cell subpopulations but different immune phenotypes have inconsistent roles in disease (6). As research on immune cells continues to deepen, the functional heterogeneity among subsets of the same immune cells has gradually been discovered by researchers; in order to achieve better therapeutic outcomes, people have begun to explore the changes in the roles of immune cells with different immunophenotypes. Many recent studies have found that the same immune cells with different phenotypes have inconsistent roles in diseases. For example, in patients with plasma cell hepatitis, significant phenotypic heterogeneity of regulatory B cells (Bregs) was observed in the blood, spleen, and tonsils. Two main suppressive Breg subpopulations, CD24hiCD38hi transitional B cells (Trb) and CD24hiCD27+ human equivalent of B10 cells, also exhibit differences in immune suppressive function, with the latter expressing more IL-10, TGF-β, and granzyme B, resulting in better inhibition of CD4+ T-cell immune responses. In addition, compared with CD24hiCD38hi Trb, CD24hiCD27+ human equivalent of B10 cells also express more CD49d and CD11a, further indicating that the two subpopulations may primarily function in different tissues (7). As mentioned above, Tregs are well-known immune suppressive cells, but with in-depth research on cell phenotypes, people have begun to discover that Tregs may not only have immune suppressive functions. Bin and his team found that Helios- Foxp3+ Tregs can secrete pro-inflammatory factors such as IFN-γ, IL-2, and IL-17, which is significantly different from the functions of Helios+ Foxp3+ Tregs (8). Similarly, Alvarez and his team found that overexpression of IL-1 receptor (IL1R1) in Tregs leads to a decrease in Foxp3 expression and promotes Tregs to shift towards the Th17 phenotype, becoming pro-inflammatory Treg cells (9). It can be seen that the same subpopulation of immune cells with different phenotypes play different or even opposite cellular functions. Clarifying the functional differences between different phenotypes of immune cells can better serve clinical treatment, adapting to the needs of precision medicine in recent years.

Thanks to the advancement of technologies such as flow cytometry and single-cell RNA analysis, researchers can delve deeper into the functional differences of immune cells with different phenotypes, providing new theoretical guidance for further exploration of disease pathogenesis, development of corresponding targeted drugs, and discovery of new biomarkers. To date, the role of immune cells with different phenotypes in AAD has been less studied, and due to the limitations of traditional analysis methods (potential confounding factors and reverse causality), the correlation between different phenotype immune cells and AAD remains unclear. Mendelian randomization is an analytical method based on the random allocation of genes to infer causal relationships in epidemiology. It can greatly avoid the interference of confounding factors and intuitively provide evidence of causal relationships between exposure and outcome (10); therefore, our team used two-sample Mendelian randomization (TSMR) analysis to estimate the causal relationship between immune cells of different phenotypes and AAD, with the aim of providing potential theoretical foundations for clinical treatment and research.

## 2 Materials and methods

### 2.1 Study design

**Figure 1:**
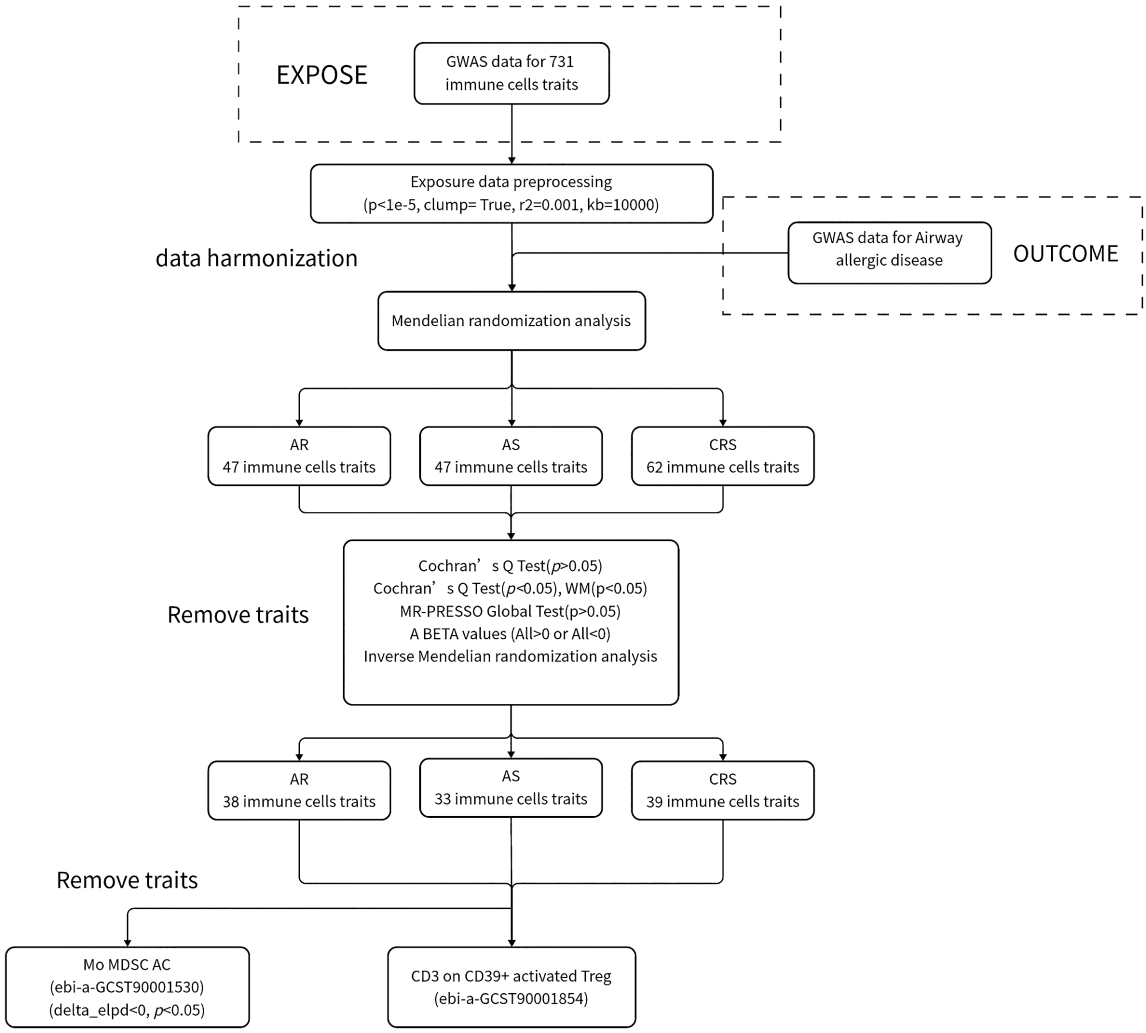
Research flowchart.

We used TSMR as a basis for assessing the causal association of 731 immune cells with AR, AS, and CRS; the use of single nucleotide polymorphisms (SNPs) as instrumental variables (IVs) and analysis of causality between exposure and outcome using MR must satisfy the following points: (I) IV is directly related to exposure; (II) IVs are not associated with any known or unknown confounding factors; and (III) In addition to influencing outcomes through exposure, IVs do not influence outcomes through any other pathways or directly. The flowchart of the specific study is shown in the figure (**Figure 1**).

### 2.2 Airway allergic disease data sources

The data for the AAD genome-wide association study were obtained from FinnGen (www.finngen.fi/en), a large Finnish biological database which launched in Finland in 2017; it brings together genomic meta-analyses of hundreds of thousands of people from universities, hospitals, biobanks, and so forth in Finland and combines information from the Finnish Health Registry population. The Genome-Wide Association Studies (GWAS) data for AR, AS, and CRS are all Finngen R9 version (https://r9.risteys.finngen.fi/), little overlap with education or any intermediary GWAS to ensure the lowest possible Type I error rate (11). The AR-GWAS data contained a total of 370,158 Finnish individuals (*N* case = 11,009, *N* control = 359,149). The AS-GWAS data contain a total of 219,753 Finnish individuals (*N* case = 9,631, *N* control = 210,122). The CRS-GWAS data contained a total of 299,737 Finnish individuals (*N* case = 16,395, *N* control = 283,342). The diagnostic criteria for all three diseases are based on the international code of diseases (12).

### 2.3 Immune cell data sources

GWAS data for 731 immune cells with different immune phenotypes from a sequence of 3,757 individual European data from Sardinia, Italy, these immune cell-associated genetic variants were identified by a reference panel based on 3,514 individuals in the Sardinian sequence or after analyzing nearly 20 million SNPs by high-density arrays, subsequently adjusted for gender, age, age squared (13), and the immune cell populations were preliminarily divided based on staining with seven major flow cytometry panels (B cell panel, Dendritic Cell (DC) panel, Maturation stages of T-cell panel, Monocyte panel, Myeloid cell panel, T lymphocytes, B lymphocytes, and Natural Killer cells (TBNK) panel, Regulatory T cell (Treg) panel). After the initial classification of cell types, the immune cells are further categorized into more detailed functional classifications based on the expression of other phenotypes on the cells. For instance, Treg cells (CD25hi CD127lo) identified in the Treg panel were further subdivided into activated Treg (CD25+++CD45RA−), resting Treg (CD25++CD45RA+), and secreting Treg (CD25++CD45RA−) (for more details, please refer to reference 13). GWAS statistics for each immune cell population are available from the GWAS Catalog (GCST90001391-GCST90002121).

### 2.4 IVs selection

To minimize the influence of weak instrumental variables, all SNPs are assessed with the *F*-statistic for strength; those with *F* > 10 are deemed robust enough for MR analysis. When using AAD as exposure, SNP threshold selected for significant correlation with exposure was set at *p* < 5 × 10^−8^ (AR = 386, CRS = 2,425, AS = 4,840, after sieving), when using immune cells as exposure, due to the challenges faced by most immune cell GWAS in achieving genome-wide significance levels (*p* < 5 × 10^−8^), the threshold for significance for IVs was established at *p* < 1 × 10^−5^. SNPs in a state of linkage disequilibrium from the remaining SNPs were excluded as tools for further analysis to ensure the independence between the selected SNPs (*r* ^2^ < 0.001, distance threshold > 10000 kb).

### 2.5 Statistical analysis

All statistical analyses were performed in R 4.3.1 software (http://www.Rproject.org).

Inverse variance weighted (IVW) was performed to assess the causal relationship between immune cells of different immunophenotypes and AAD (14), mainly using the “TwoSampleMR” package. IVW is the primary reference index for MR analysis, assuming all SNPs serve as valid instrumental variables without horizontal pleiotropy, thus delivering stable and valid causal estimates based on this. The IVW is prone to bias due to the difficulty in practical application to achieve complete absence of horizontal pleiotropy in the included SNPs. Considering the above, the MR-Egger method and the weighted median method were used to complement the results of the IVW method. The MR-Egger method allows for the presence of (intercept term) pleiotropy, allowing for IVs that are all invalid, thus identifying potential pleiotropic bias, but with poor precision. The weighted median method (WM) selects the median for the overall MR estimation, particularly when 50% of the weights are derived from valid IVs, MR-Egger can still make causal effect inferences consistent with IVW and with similar efficiency (15). If the null hypothesis (*p* < 0.05) is rejected, the Cochran’s *Q* test (*Q p*-value) is used to include heterogeneity between SNPs, heterogeneity was considered to be present, and the results were evaluated using the WM (13). When the results of the WM method are also not significant, it indicates that there is no causal relationship between the exposure and the outcome, and it is considered negative and removed. To remove the effect of horizontal pleiotropy on the results, we used MR-PRESSO to assess whether the results were pleiotropic or not (16), direct exclusion of causal inference effects if there is pleiotropy. When the same exposure exists for three allergic diseases, if there is pleiotropy, the “cause” package is used to determine whether the pleiotropy affects the stability of causal inference, the model with a fixed causality of zero (sharing model) is compared with the model with a causal relationship (causal model), and the expected log pointwise posterior density (ELPD) is used to assess the degree of fit of the two models (delta_ELPD = ELPDsharing - ELPDcasual). If delta_ELPD is less than 0, the causal model is better than sharing model, conversely, the sharing model outperforms the causal model, subsequently further looking at the *p*-value. When there is significance in the *p*-value (*p* < 0.05) and delta_ELPD is negative, it can be assumed that horizontal multiplicity does not affect the inference of causal effects (17). In addition, we also used scatterplots to assess causality, which can visualize the heterogeneity of all causal assessment methods and show whether outliers have a large impact on the results; funnel plots and leave-one-out methods were used to evaluate the sensitivity and stability of the results.

## 3 Results

### 3.1 Causal analysis of immunophenotypes and airway allergic diseases

To investigate the effect of different immune cell phenotypes on AADs, we performed TSMR on 731 immune cell phenotypes in relation to AR, AS, and CRS, respectively, with IVW as the primary analytical method, while the WM method was applied in cases of substantial heterogeneity. A *p*-value of less than 0.05 indicated a significant causal relationship. All IVs included in the final analysis had *F*-values greater than 10, and all results were tested for stability by funnel plots and leave-one-out tests.

### 3.2 Allergic rhinitis

**Figure 2:**
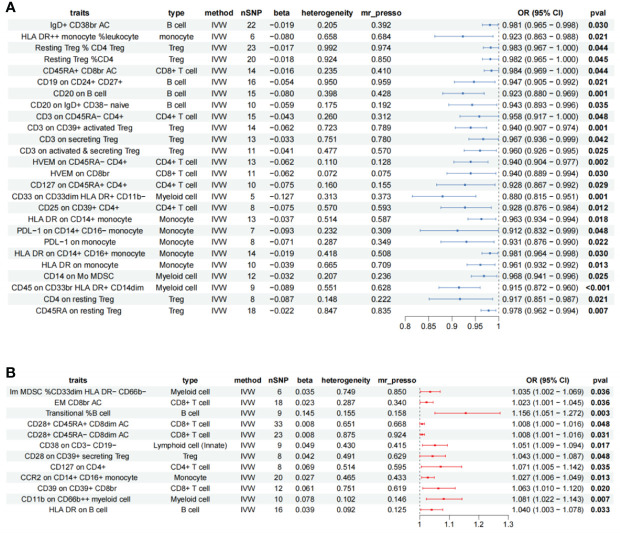
Forest plot showing all immune cells with a positive causal relationship with AR. **(A)** immune cells with pathogenic effects. **(B)** Immune cells with protective effects; CI, confidence interval; br, bright cell; %, relative count; AC, absolute count; IVW, inverse variance weighted.

A total of 47 immune cell phenotypes with clearly significant IVW were identified in the AR; all of them were free of heterogeneity. We excluded Mo MDSC AC (absolute count) due to horizontal pleiotropy (MR-PRESSO *p* = 0.044). Scatter plots were taken to see if there was a difference in the trends of the 46 immune cell analysis methods (beta), and five immune cells were markedly different, unstable for causal inference, and were removed. Reverse Mendelian randomization was then performed to explore whether there was reverse causality between AR and the remaining 41 immune cells, with AR as the exposure and 731 immune cells as the endpoints. The analysis revealed bidirectional causality for CD4 on CD39+ activated Treg, FSC-A on CD8br (bright), and CD45 on lymphocyte, which were excluded. The final screen identified 38 immunophenotypes of immune cells (**Supplementary Table S1**), 12 different phenotypes of immune cells as risk factors for AR. Immune cells of 26 different phenotypes are protective against AR (**Figure 2**).

### 3.3 Allergic asthma

**Figure 3:**
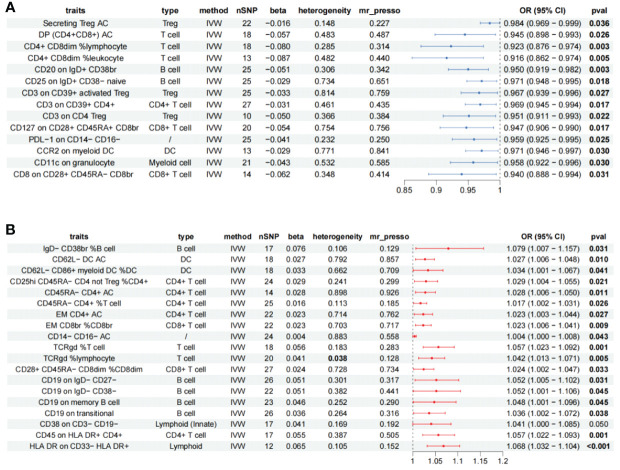
Forest plot showing all immune cells with a positive causal relationship with AS. **(A)** immune cells with protective effects. **(B)** Immune cells with protective effects; CI, confidence interval; br, bright cell; %, relative count; AC, absolute count; IVW, inverse variance weighted; DP, double positive; EM, effector memory.

A total of 47 different cellular immunophenotypes significantly associated with AS were identified, among which 10 were removed due to significant heterogeneity. Despite the presence of heterogeneity in HVEM on CD45RA− CD4+ (Cochran’s Q *p* = 0.017, WM = 0.018), HLA DR on plasmacytoid DC (Cochran’s Q *p* < 0.001, WM < 0.001), and HLA DR on DC (Cochran’s Q *p* < 0.001, WM < 0.001), the results of the WM method were statistically significant and thus retained. CD20 on IgD− CD27− (beta^MR-Egger^ = −0.016, beta^WM^ = 0.014, beta^IVW^ = 0.067) and CD14 on CD33dim HLA DR+ CD11b+ (beta^MR-Egger^ = -0.016, beta^WM^ = 0.022, beta^IVW^ = 0.040) were removed due to inconsistent trends in analytical results, HVEM on CD45RA− CD4+ (MR-PRESSO *p* = 0.029), HLA DR on plasma cell DCs (MR-PRESSO *p* = 0.002), HLA DR on DCs (MR-PRESSO *p* = 0.017) significant levels of multiplicity were present and were excluded, reverse Mendelian results suggesting bidirectional causation of FSC-A on CD8br, CD4 on secreting Treg were eliminated, resulting in 33 remaining immune cells (**Supplementary Table S2**), 19 immune cells are risk factors for AS, and 14 immune cells as protective factors in AS (**Figure 3**).

### 3.4 Chronic sinusitis

**Figure 4:**
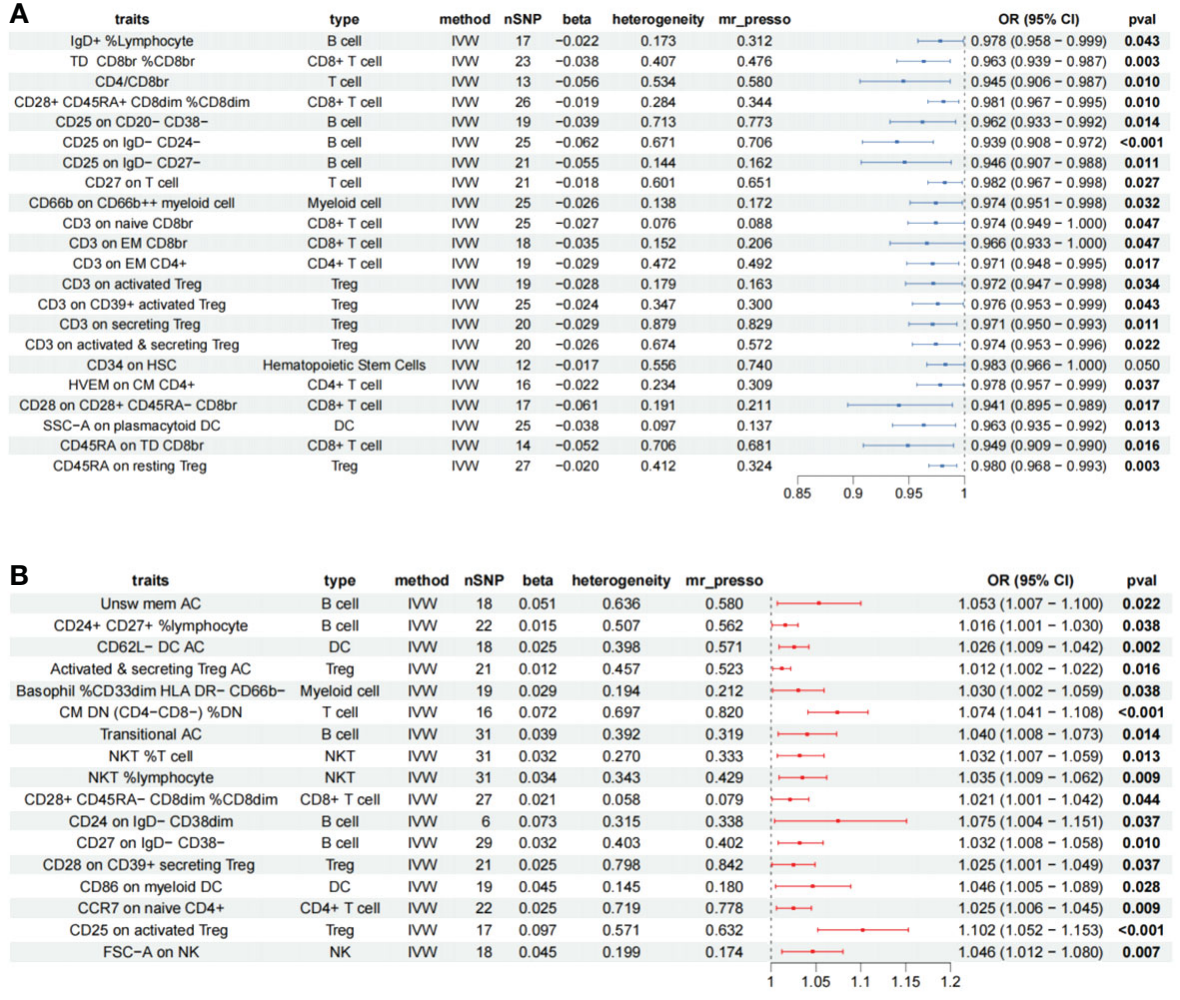
Forest plot showing all immune cells with a positive causal relationship with CRS. **(A)** immune cells with protective effects. **(B)** Immune cells with protective effects; CI, confidence interval; br, bright cell; %, relative count; DN, double negative; DC, dendritic cell; HSC, hematopoietic stem cells; %, relative count; AC, absolute count; IVW: inverse variance weighted; TD, terminally differentiated; CM, central memory; DN, double negative.

A total of 62 immune cells with different immunophenotypes associated with chronic rhinosinusitis were identified, of which 19 were heterogeneous, and 10 immune cells with significant WM results such as CM CD4+ AC (Cochran’s Q *p* < 0.001, WM = 0.024), CD4+ AC(Q *p* < 0.001, WM = 0.017) were retained, excluding nine highly heterogeneous immune cells; scatter plots to observe the trend of results for the remaining 53 immune cells, CD127− CD8br %T cell (beta^MR-Egger^ = −0.003, beta^WM^ = 0.028, beta^IVW^ = 0.056), CD27 on IgD- CD38br (beta^MR-Egger^ = −0.097, beta^WM^ = 0.071, beta^IVW^ = 0.068) had inconsistent trends; the inference of causality was not robust. Further assessment was performed for the presence of horizontal pleiotropy among the remaining immune cells. A total of 12 immune cells such as CM CD4+ AC (MR-PRESSO *p* = 0.001) and CD4+ AC (MR-PRESSO *p* = 0.001) were excluded due to the presence of horizontal pleiotropy. Subsequently, reverse Mendelian randomization of the remaining immune cells did not reveal the existence of significant reverse causality and, finally, 39 immune cells remained (**Supplementary Table S3**), 17 immune cells are risk factors for CRS, and 22 immune cells are protective factors for CRS (**Figure 4**).

### 3.5 Causal analysis of immune cells and AAD

**Figure 5:**
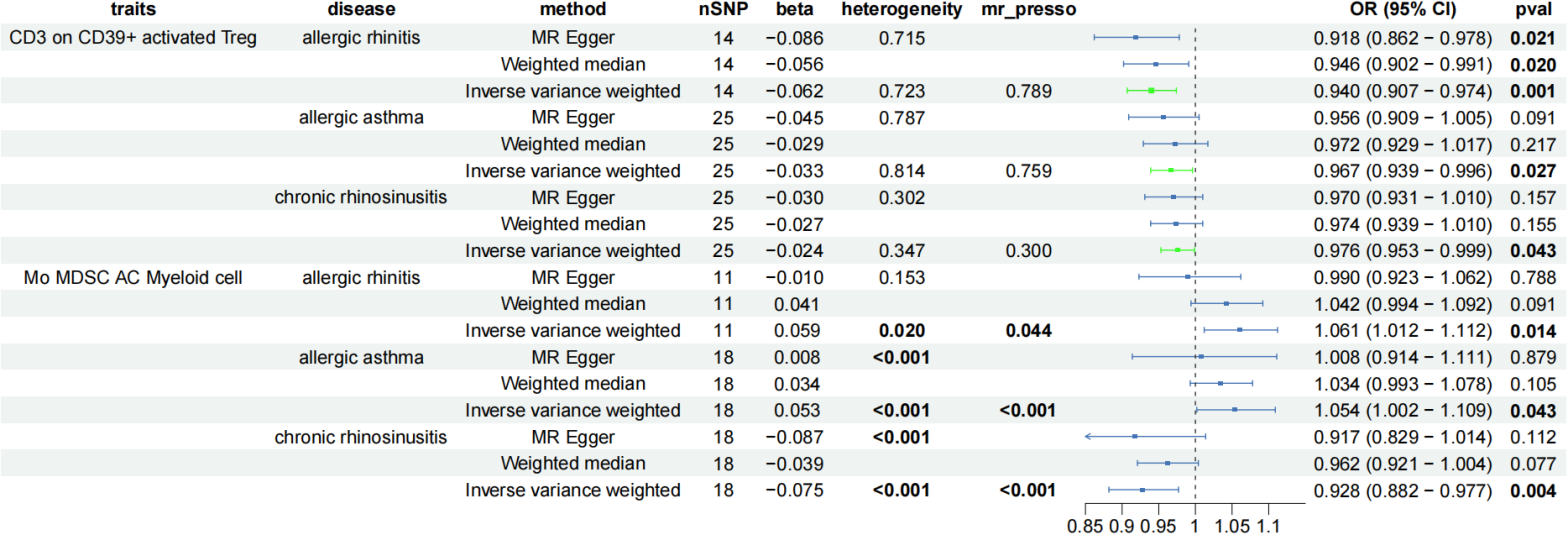
Forest plot showing the immune cells with positive causal relationship with three AAD; CI, confidence interval; AC, absolute cell counts.

To explore the immune cell phenotypes that are most likely to have an impact on AADs, three immune cell populations significantly associated with AADs, AS, AR, and CRS were taken for intersectional processing, and CD3 on CD39+ activated Treg was the only immune cell type that had positive causality for all three different AADs (IVW^AR^ = 0.001, IVW^CRS^ = 0.043, IVW^AS^ = 0.027) and was protective in all three diseases (OR^AR^ = 0.940, OR^AS^ = 0.967, OR^CRS^ = 0.976) without heterogeneity or horizontal pleiotropy (**Figure 5**).

When the screening conditions were relaxed moderately (considering only the presence or absence of significance of IVW, heterogeneity and trend inconsistency were acceptable), Mo MDSC AC (Myeloid cell, IVW^AR^ = 0.014, IVW^AS^ = 0.043, IVW^CRS^ = 0.004) appeared to be a potentially critical immune cell phenotype for AADs. Mo MDSC AC is pleiotropic in all three diseases, and to determine whether pleiotropy actually affects the inference of causal effects, we validated it using the CAUSE package (**Figure 5**). The results suggest that horizontal pleiotropy of the three disease species can influence the inference of causality, and no causality exists between Mo MDSC AC and AAD (**Table 1**).

**Table 1:** Cause method of determining the effect of horizontal pleiotropy on causality.

disease	elpd of causal	elpd of sharing	delta elpd	*z*	*p*-value
AR	−1.10	−0.67	0.43	1.1	0.85
AS	−1.30	−0.58	0.72	5.3	1.00
CRS	−0.41	−0.57	−0.16	−0.28	0.39

## 4 Discussion

Building on Finland’s publicly available biogenetic data, we investigated potential causal relationships between a total of 731 phenotypes of immune cells and AR, AS, and CRS. A total of 101 immune cells showed positive causality for AR, AS, and CRS (AR = 38, AS = 33, CRS = 39), involving seven immune cell populations.

Classical Treg is a subpopulation of CD4+ T cells producing FOXP3 and CD25, with immunosuppressive functions, which could be further categorized into natural Treg (nTreg) and induced Treg (iTreg), with nTreg develop directly derived from the thymus, whereas iTreg are mainly produced by naïve CD4+ T cells after peripheral antigen stimulation and can differentiate into iTreg of different phenotypes (18), both of which require Foxp3 to maintain functional stability. Treg performs its immunosuppressive function by secreting inhibitory cytokines such as IL-10, TGF-β, IL35, and perforin. Classical Tregs with immunosuppressive functions are often characterized as CD4+ CD25+ CD127- Foxp3+ Treg cells. CD127 is also a key phenotype used to distinguish Tregs from other types of CD4+ T cells, and the expression of CD127 is negatively correlated with the expression of Foxp3 (19). When Treg function is suppressed, it can easily lead to the onset and exacerbation of many autoimmune diseases (20). In addition to Th1/Th2 imbalance, Treg/Th17 is an important influence on the pathogenesis of airway allergic inflammation (21, 22); there is a very complex link between Th17 and Treg, which both diverge from naïve CD4+ T cells, which can differentiate into Treg only when induced by TGF-β. When TGF-β and IL-6 are present at the same time, the CD4^+^ T cells will turn to differentiate into Th17, this process is in a state of dynamic equilibrium, which maintains a stable immune state of the organism, when there is an imbalance of the cytokine environment in the surroundings, the production of Treg decreases, and the production of Th17 increases, which aggravate the allergic inflammation and the high reaction of the airways (23, 24). In conclusion, classical Treg is important for keeping immune stability and suppressing the onset of autoimmune diseases. Our study found that CD3 on CD39+-activated Tregs (CD4+ CD39+ CD25+++ CD45RA- CD127^lo^ Treg) played a protective role in three types of AAD. CD39 has been identified as an E-NTPD enzyme, predominantly expressed on the surface of Treg cells, which can degrade extracellular ATP into AMP, thereby reducing the pro-inflammatory effects of extracellular ATP and enhancing the immunosuppressive effects of Tregs (25, 26). Aberrant CD39 expression can suppress the function of Tregs, increase the expression of sensitized CD8+ T cells, leading to increased allergic symptoms, and can also alter the distribution of NKT in the lungs of mice, exacerbating AAD (27, 28). Similarly, a significant decrease in the proportion of CD3+ CD39+ Tregs was also observed in the peripheral blood of patients with severe asthma. In addition, in recent years, CD39 has also been used to identify and recognize high-functioning Tregs (29). The above evidence proves the importance of CD39+ for the immunosuppressive function of Tregs. CD3+ is a phenotype shared by T cells, which can promote the activation and differentiation of Tregs and enhance their immunosuppressive function. During the activation process of T cells, the CD3-TCR complex must first bind to antigenic peptide fragments presented by MHC on the surface of other antigen-presenting cells, subsequently activating the proliferation and inhibitory functions of T cells. Once T cells are activated, to limit the intensity of T-cell activation, cells achieve the recycling and degradation of the CD-TCR complex through endosomal recycling (30). This self-regulatory process occurs in almost all T cells, including Tregs. Therefore, the expression level of CD3 determines the quantity of CD3-TCR and the surface density level of endosomal recycling, which also represents the intensity of T-cell activation to a certain extent. Treatment with anti-CD3 can significantly inhibit the proliferation of CD4+ T cells in the peripheral blood of patients with AS and, in mice, anti-CD3 treatment was observed to inhibit CD4+ T-cell activity in lung tissue and blood vessels, significantly reducing the level of asthma inflammation (31). Although there are few reports on the relationship between CD3 expression levels and Treg function and activation, the above evidence also provides us with certain insights, that is, an increase in CD3 expression levels may help enhance the immunosuppressive function of Tregs and promote Treg activation. Our other Treg results also suggest that the expression level of CD3 on different phenotypic Tregs may be key to Treg treatment of AAD (**Figures 2**–**4**). In summary, our research results further emphasize the importance of CD3 on CD39+-activated Tregs in the development of AADs from a causal perspective and provide new insights and a basis for further exploration of new AAD treatment targets based on CD3 and CD39 in the future.

However, not all Treg in all states have immunosuppressive functions. CD4+ CD25+ Treg isolated from patients with SLE were unable to inhibit effector T cells and monocytes from releasing inflammatory cytokines, although they remained cellularly active, probably because tumor necrosis factor α (TNF-α) causes Treg to impair the function of Treg (32, 33). In addition, Treg can also express inflammatory cytokines from other types of Th cells through internal reprogramming, a behavior known as Treg plasticity, which has been identified in multiple autoimmune diseases (23, 34), This Th2-like Treg cell production was observed in the sensitized state, the Th2-like Treg is triggered by IL-4 and IL-13 and is associated with significant cellular phenotypic changes, which can secrete a large number of sensitizing pro-inflammatory factors, but still have some inhibitory activity of Th1 and Th2 (35). The above evidence suggests that Treg does not simply exercise an immunosuppressive function, but may also contribute to disease exacerbation in different environments and with different phenotypes. Human CD4+ Foxp3+ T cells can generally be classified into Foxp3^lo^ CD25++ CD45RA+ (resting Treg), Foxp3^hi^ CD25+++ CD45RA− (activated Treg), and Foxp3^lo^ CD25++ CD45RA− (cytokine-secreting T cells). Unlike resting and activated Tregs, Foxp3^lo^ CD25++ CD45RA− cytokine-secreting T cells do not have an immunosuppressive function but can exacerbate inflammation by secreting cytokines such as IL-17 and IFN-γ (36). Some scholars currently consider it to be a subtype of Tregs, and this type of Cytokine-Secreting Treg has been observed to have significantly higher expression in the peripheral blood of asthma patients compared to healthy individuals, with expression levels increasing as the severity of asthma increases (37). Our research did not identify any specific Treg phenotypes that promote AS. However, we noted that the secreting Treg AC (CD25++ CD45RA- CD127lo) have a protective effect against AS. The primary distinction in phenotype between it and cytokine-secreting T cells lies in the opposite levels of expression of CD127 and Foxp3, highlighting to some extent the importance of Foxp3 and CD127 expression levels in maintaining the suppressive function of Tregs. Furthermore, in AR, we found that CD28 on CD39+ secreting Treg (CD28 on CD4+ CD39+ CD25++ CD45RA- CD127^lo^) has a pathogenic role. CD28 is a protein receptor expressed on the surface of T cells that can bind to CD80/CD86 to activate T cells, and CD28 plays an important role in maintaining Treg proliferation and homeostasis (38). However, Zheng and his team have found that using anti-CD28 antibodies to block CD28 can enhance the inhibitory function of Tregs, possibly due to the blockade of CD28 altering the interaction between Tregs and dendritic cells (DCs) (39). Although our findings suggest that CD28 on CD39+ secreting Treg may be a risk factor for AR, the absence of literature directly linking this specific phenotype with AR, together with the potential risk of false positives due to the selection of analysis thresholds in our study, necessitates further confirmation through clinical and cellular experimentation. The role of Tregs in CRS remains complex, with studies reporting that CD25+/CD4+ FOXP3+ T cells are significantly higher in CRS with nasal polyps than in CRS without nasal polyps. However, reports also indicate that Treg dysfunction can lead to Th1/Th2 imbalance, exacerbating CRS (40, 41). Moreover, the immunological process of CRS varies among different ethnicities and regions; for instance, patients with CRS with nasal polyps in Western countries tend to have Th2-type inflammation, while patients in Southeast Asia tend to have Th1/Th17 immune imbalance (42). Among protective factors, we have found that the expression level of CD3 significantly affects the protective role of Tregs in CRS. Our research also discovered that activated and secreting Treg AC is the only Treg phenotype combination that has a pathogenic effect on CRS. Consistent with our findings, Ickrath and his team discovered that the expression of activated Treg in nasal polyp tissues of chronic rhinosinusitis is significantly higher than in peripheral blood (43). Tregs have a strong suppressive effect on inflammation, and we consider that the main reason for the dysfunction of Tregs in CRS may be the functional plasticity of Tregs. In summary, this provides important insights for exploring the functional diversity of Tregs in CRS and innovation in future treatment strategies.

B cells develop by differentiation from bone marrow hematopoietic stem cells and are most well known for their function of producing antibodies. B cells are very important to the immune system and are the primary effector cells of humoral immunity. B cells are categorized into different subpopulations based on their function and origin, which includes naive B cells, transitional B cells, plasma cells, and others. Similarly, immune phenotypes and functions differ between subtypes (44). It has been established that B cells can act and activate Th2 cells as antigen-presenting cells. Significantly elevated nasal mucosal and blood species–specific IgE antibodies were observed in AR patients, expression of CD23+ B cells was markedly elevated in AR patients in comparison to normal subjects (45). A recent study discovered that CD38+ B cells cause Treg/Th17 imbalance and exacerbate allergic symptoms through the secretion of IL-6, and that the combined application of CD38-antibody may enhance the therapeutic effect of AIT (46). Apart from the sensitizing effect through IgE, Breg is a subpopulation of B cells involved in modulating immune tolerance, promotion of naive CD4+ T-cell differentiation into Treg cells, and suppression of Th1 and Th17 cell differentiation (47); as a major IL-10 secreting cell, Breg can effectively inhibit allergic inflammation and suppress eosinophil production; as with Treg described earlier, there has been insufficient study of the function of Breg in AAD, which may be due in part to its different immunophenotypes (48). Trb are immature B cell types in transition that have just acquired a functional B-cell antigen receptor and from the bone marrow; it migrates to the peripheral lymphoid organs, awaiting activation and maturation. Different subtypes of Transitional B cells also have different functions, for example, the T2/T3 type produces IL-10 to reduce CD4^+^ T-cell proliferation but, in some autoimmune diseases, Trb also secretes anti-inflammatory factors (IL-6 and TNF-α) that disrupt the Treg/Th17 balance (49, 50). Our results suggest that CD19 on transitional may be a risk factor for AS; however, a recent study found that after 6 months of treatment with Dupilumab in patients with severe asthma, the number of TrB cells in the patients was elevated and the total serum IgE was significantly decreased, but the paper did not explore the specific functions and immunophenotypes of TrB; this difference may also be due to different immunophenotypes (51), in addition, in AR, the relative count of Trb may be a potential risk factor (Transitional %B cell, *p* ^IVW^ = 0.003, OR=1.156), whereas the absolute count (AC) of Trb may be a risk factor for being a CRS (Transitional AC, *p* ^IVW^ = 0.014, OR = 1.04).

Maturation stages of T cells mainly include CD4+/CD8+ T cells. CD4+ T cells, CD45RA is primarily expressed on the surface of naïve T cells and is one of their surface markers. The expression of CD45RA gradually decreases during the maturation process of T cells (52), also known as Th0 cells, can differentiate into different subtypes such as Th1, Th2, Th3, Treg, Th17, and Tfh. CD8+ T cells are also known as cytotoxic T cells, and their special antiviral and antitumor capabilities are well recognized. Different subtypes have different impacts on the immune system; different T cells cooperate with each other through promotion or suppression to coordinate the immune balance in the body. Myeloid cells include granulocytes, monocytes, dendritic cells, and other cells (53). Myeloid-derived suppressor cells (MDSCs) consist of myeloid-derived myeloid progenitor cells and immature cells; MDSC expression was markedly increased in acute inflammation, severe infections and tumors, and negatively regulates immune function, a recent study showed that MDSCs are engaged in the invigoration of Th2 cells, which induces airway hyperresponsiveness and asthma, and MDSC levels have been reported in AR, AS, and other allergic diseases. Elevated MDSC is reported in AR, AS, and other allergic diseases, but MDSC expression is also elevated in healthy individuals after exposure to allergens, suggesting the possibility that MDSCs may have both inhibitory and pro-validating roles, and that the roles of different phenotypes of MDSCs and chemokines may be the key factors influencing the function of MDSCs (54).Our research only found that one MDSC phenotype has a protective effect against AAD (CD14 on Mo MDSC, *p* ^IVW-AR^ = 0.025, beta^AR^ = -0.032, OR^AR^ = 0.968). No other causal relationship between MDSCs and AAD was identified.

Monocytes are also an important part in the immune system. The most studied monocyte in AAD is the macrophage, which can be polarized by stimuli from the surrounding environment into subpopulations with different phenotypes and functions (55), for example, M2-type macrophages can promote Th2 cell differentiation, leading to Th1/Th2 imbalance and recruitment of eosinophilic infiltration, leading to exacerbation of AAD symptoms. It is worth noting that no monocyte type was found to have a direct causal relationship with CRS in our results, in addition; cDC are important immune cells with strong antigen phagocytosis and can promote Treg differentiation. It has also been shown that cDC can exacerbate AAD by modulating Th2 cells through the p38α and Fas pathways (56–58); however, in our results, no cDC cells were found to have a positive causal relationship with AR. It is noteworthy that some studies have found that high expression of CD62L in plasmacytoid dendritic cells of AS patients can promote Th2-type inflammatory responses (59). Our research has discovered that low expression of CD62L in cDCs can also promote AS. This result suggests that even the same phenotype may have different effects on different types of DC cells, and the expression and function of CD62L may be crucial in DCs and AAD.

## 5 Conclusion

To our knowledge, it is the first research to explore the causal relationship between immune cells of different immunophenotypes and AAD; we demonstrated the causal relationship between the three AAD disorders and different phenotypes of immune cells through a rigorous two-way two-sample Mendelian randomization analysis, which reduces confounding bias and improves the reliability of our results. As our results demonstrate, different phenotypes of immune cells have different effects on AAD, and perhaps there is a difference in just a single phenotype, but the functions may be completely opposite, suggesting a complex regulatory model between the immune system and AAD. In addition, our study results provide a theoretical foundation for the development of more personalized and precise treatment plans for AAD and can also help clinicians better understand the pathophysiological mechanisms of patients and formulate more effective treatment strategies. By gaining a deeper understanding of the interactions between immune cells and AAD, we can further explore new therapeutic targets. Although further research is needed to validate our results and explore their clinical application value, we believe that this study will provide valuable reference and guidance for the prevention and treatment of AAD.

However, some limitations of our study exist, firstly, we appropriately expanded the inclusion when immune cells were used as exposure (*p* < 1 × 10^−5^), which may somewhat contribute to biased results and false positives; second, all our data sources are based on European populations, and extrapolation to other races should be construed with caution and, last, because of the lack of personal information, we were unable to stratify the population analysis any further. Additionally, it is regrettable that there is still a lack of expression Quantitative Trait Loci (eQTL) data for different phenotype immune cells in publicly available databases, which to some extent limits our ability to better understand and explore the role of different phenotype immune cells in AAD. Therefore, we will continue to focus on and explore the eQTL data of different phenotype immune cells in our future research to improve our study results. We believe that with the continuous improvement of public databases and the efforts of more researchers, the role of different phenotype immune cells in AAD will be better understood in the future, and new biomarkers and therapeutic targets will be discovered.

In conclusion, we expect that this study will help researchers to explore the role of different immunophenotypes of immune cells for AAD and provide valuable clues for the development of further immunotherapeutic approaches for AAD.

## Data availability statement

The original contributions presented in the study are included in the article/**Supplementary Material**. Further inquiries can be directed to the corresponding author.

## Ethics statement

The studies involving humans were approved by Sardinian Regional Ethics Committee The Coordinating Ethics Committee of the Hospital District of Helsinki and Uusimaa (HUS). The studies were conducted in accordance with the local legislation and institutional requirements. Written informed consent for participation was not required from the participants or the participants’ legal guardians/next of kin in accordance with the national legislation and institutional requirements.

## Author contributions

ZX: Conceptualization, Data curation, Formal analysis, Methodology, Project administration, Resources, Software, Validation, Visualization, Writing – original draft, Writing – review & editing. RL: Conceptualization, Data curation, Formal analysis, Methodology, Resources, Software, Validation, Visualization, Writing – review & editing. LW: Data curation, Formal analysis, Investigation, Methodology, Resources, Software, Validation, Writing – review & editing. YiW: Data curation, Methodology, Resources, Writing – review & editing. YT: Data curation, Writing – review & editing. YS: Data curation, Writing – review & editing. YM: Data curation, Writing – review & editing. RL: Data curation, Writing – review & editing. YaW: Data curation, Writing – review & editing. CZ: Data curation, Writing – review & editing. SH: Data curation, Writing – review & editing. SD: Data curation, Writing – review & editing. HP: Data curation, Writing – review & editing. JX: Conceptualization, Data curation, Formal analysis, Funding acquisition, Investigation, Methodology, Project administration, Resources, Software, Supervision, Validation, Visualization, Writing – review & editing.
