# Supplementary material for: Pathogenic role of different phenotypes of immune cells in airway allergic diseases: a study based on Mendelian randomization
Source: Front Immunol. 2024 May 15;15:1349470. doi: 10.3389/fimmu.2024.1349470 (PMC11133742; doi:10.3389/fimmu.2024.1349470)
Supplement: Supplementary file 1 [file DataSheet_1.pdf]

STROBE-MR checklist of recommended items to address in reports of Mendelian randomization studies[1 2]

Item No.	Section		Checklist item	Page No.	Relevant text from manuscript
1	**TITLE and ABSTRACT**		Indicate Mendelian randomization (MR) as the study's design in the title and/or the abstract if that is a main purpose of the study	01	Pathogenic role of different phenotypes of immune cells in airway allergic diseases: a study based on Mendelian randomization (MR)
	**INTRODUCTION**				
2	**Background**		Explain the scientific background and rationale for the reported study. What is the exposure? Is a potential causal relationship between exposure and outcome plausible? Justify why MR is a helpful method to address the study question	02	Different subsets of the same immune cells have different functions, such as T cells, which have the potential to differentiate into CD4+ T cells and CD8+ T cells...... the correlation between different phenotype immune cells and AAD remains unclear. Mendelian randomization is an analytical method based on the random allocation of genes to infer causal relationships in epidemiology. It can greatly avoid the interference of confounding factors and intuitively provide evidence of causal relationships between exposure and outcome (10), therefore, our team used two-sample mendelian randomization (TSMR) analysis to estimate the causal relationship between immune cells of different phenotypes and AAD, with the aim of providing potential theoretical foundations for clinical treatment and research.
3	**Objectives**		State specific objectives clearly, including pre-specified causal hypotheses (if any). State that MR is a method that, under specific assumptions, intends to estimate causal effects	03	our team used two-sample mendelian randomization (TSMR) analysis to estimate the causal relationship between immune cells of different phenotypes and AAD, with the aim of providing potential theoretical foundations for clinical treatment and research.
	**METHODS**				
4	**Study design and data sources**		Present key elements of the study design early in the article. Consider including a table listing sources of data for all phases of the study. For each data source contributing to the analysis, describe the following:		
		a)	Setting: Describe the study design and the underlying population, if possible. Describe the setting, locations, and relevant dates, including periods of recruitment,	03	The data for the AAD genome-wide association study were obtained from FinnGen

	exposure, follow-up, and data collection, when available.		(www.finngen.fi/en), a large Finnish biological database which Launched in Finland in 2017, it brings together genomic meta-analyses of hundreds of thousands of people from universities, hospitals, biobanks, etc. in Finland and combines information from the Finnish Health Registry population. The GWAS data for AR, AS, and CRS are all Finngen R9 version(https://r9.risteys.finngen.fi/), little overlap with education or any intermediary GWAS to ensure the lowest possible Type I error rate (11). The AR-GWAS data contained a total of 370,158 Finnish individuals(N case=11009, N control=359149), The AS-GWAS data contains a total of 219753 Finnish individuals(N case=9631, N control=210122), The CRS-GWAS data contained a total of 299,737 Finnish individuals(N case=16395, N control=283342), The diagnostic criteria for all three diseases are based on the international code of diseases (12).
b)	Participants: Give the eligibility criteria, and the sources and methods of selection of participants. Report the sample size, and whether any power or sample size calculations were carried out prior to the main analysis	03	a large Finnish biological database which Launched in Finland in 2017, it brings together genomic meta-analyses of hundreds of thousands of people from universities, hospitals, biobanks, etc. in Finland and combines information from the Finnish Health Registry population. The GWAS data for AR, AS, and CRS are all Finngen R9 version(https://r9.risteys.finngen.fi/), little overlap with education or any intermediary GWAS to ensure the lowest possible Type I error rate (11). The AR-GWAS data contained a total of 370,158 Finnish individuals(N case=11009, N control=359149), The AS-GWAS data contains a total of 219753 Finnish individuals(N case=9631, N control=210122), The CRS-GWAS data contained a total of 299,737 Finnish individuals(N case=16395, N control=283342), The diagnostic criteria for all three diseases are based on the international code of diseases (12).
c)	Describe measurement, quality control and selection of genetic variants	03	To minimize the influence of weak instrumental variables, all SNPs are assessed with the F-statistic for strength; those with F > 10 are deemed robust enough for MR analysis. When using AAD as exposure, SNP threshold selected for significant

					correlation with exposure was set at $p < 5 \times 10$-8 (AR = 386, CRS = 2425, AS = 4840, after sieving), when using immune cells as exposure, due to the challenges faced by most immune cell GWAS in achieving genome-wide significance levels ($p <$ 5e-8), the threshold for significance for IVs was established at $p < 1 \times 10$-5. SNPs in a state of linkage disequilibrium from the remaining SNPs were excluded as tools for further analysis to ensure the independence between the selected SNPs (r2 < 0.001, distance threshold > 10000 kb).
		d)	For each exposure, outcome, and other relevant variables, describe methods of assessment and diagnostic criteria for diseases	03	The diagnostic criteria for all three diseases are based on the international code of diseases
		e)	Provide details of ethics committee approval and participant informed consent, if relevant	-	The data used in this study were derived from previously published GWAS summary statistics, and all data were collected with the informed consent of the patients and have been approved by the local ethical authorities; therefore, no additional ethical approvals needed to be provided for this study.
5	**Assumptions**		Explicitly state the three core IV assumptions for the main analysis (relevance, independence and exclusion restriction) as well assumptions for any additional or sensitivity analysis	03	We used TSMR as a basis for assessing the causal association of 731 immune cells with AR, AS, and CRS, use of single nucleotide polymorphisms (SNPs) as instrumental variables (IVs) and Analysis of causality between exposure and outcome using MR must satisfy the following points: (Ⅰ) IVs is directly related to exposure, (Ⅱ) IVs are not associated with any known or unknown confounding factors, (Ⅲ) In addition to influencing outcomes through exposure, IVs do not influence outcomes through any other pathways or directly.
6	**Statistical methods: main analysis**		Describe statistical methods and statistics used		
		a)	Describe how quantitative variables were handled in the analyses (i.e., scale, units, model)	-	-
		b)	Describe how genetic variants were handled in the analyses and, if applicable, how their weights were selected	-	-

		c)	Describe the MR estimator (e.g. two-stage least squares, Wald ratio) and related statistics. Detail the included covariates and, in case of two-sample MR, whether the same covariate set was used for adjustment in the two samples	03	Inverse variance weighted (IVW) was performed to assess the causal relationship between immune cells of different immunophenotypes and AAD (14), mainly using the 'TwoSampleMR' package. IVW is the primary reference index for MR analysis, assuming all SNPs serve as valid instrumental variables without horizontal pleiotropy, thus delivering stable and valid causal estimates based on this. The IVW is prone to bias due to the difficulty in practical application to achieve complete absence of horizontal pleiotropy in the included SNPs.
		d)	Explain how missing data were addressed	-	-
		e)	If applicable, indicate how multiple testing was addressed	-	-
7	**Assessment of assumptions**		Describe any methods or prior knowledge used to assess the assumptions or justify their validity	03	To minimize the influence of weak instrumental variables, all SNPs are assessed with the F-statistic for strength; those with F > 10 are deemed robust enough for MR analysis.
8	**Sensitivity analyses and additional analyses**		Describe any sensitivity analyses or additional analyses performed (e.g. comparison of effect estimates from different approaches, independent replication, bias analytic techniques, validation of instruments, simulations)	04	If the null hypothesis ($p < 0.05$) is rejected, the Cochran's Q test(Q p-value) is used to include heterogeneity between SNPs, heterogeneity was considered to be present and the results were evaluated using the WM (13), when the results of the WM method are also not significant, it indicates that there is no causal relationship between the exposure and the outcome, and it is considered negative and removed. To remove the effect of horizontal pleiotropy on the results, we used MR-PRESSO to assess whether the results were pleiotropic or not (16), direct exclusion of causal inference effects if there is pleiotropy. When the same exposure exists for three allergic diseases, if there is pleiotropy, the 'cause' package is used to determine whether the pleiotropy affects the stability of causal inference, the model with a fixed causality of zero (sharing model) is compared with the model with a causal relationship (causal model), and the expected log pointwise posterior density (ELPD) is used to assess the degree of fit of the two models.
9	**Software and pre-registration**				

		a)	Name statistical software and package(s), including version and settings used	03	All statistical analyses were performed in R 4.3.1 software
		b)	State whether the study protocol and details were pre-registered (as well as when and where)	-	-
	**RESULTS**				
10	**Descriptive data**				
		a)	Report the numbers of individuals at each stage of included studies and reasons for exclusion. Consider use of a flow diagram	03	SNP threshold selected for significant correlation with exposure was set at p<5×10-8 (AR=386, CRS=2425, AS=4840, after sieving).
		b)	Report summary statistics for phenotypic exposure(s), outcome(s), and other relevant variables (e.g. means, SDs, proportions)	-	-
		c)	If the data sources include meta-analyses of previous studies, provide the assessments of heterogeneity across these studies	-	-
		d)	For two-sample MR:<br>i. Provide justification of the similarity of the genetic variant-exposure associations between the exposure and outcome samples<br>ii. Provide information on the number of individuals who overlap between the exposure and outcome studies	03	The data for the AAD genome-wide association study were obtained from FinnGen (www.finngen.fi/en), a large Finnish biological database which Launched in Finland in 2017, it brings together genomic meta-analyses of hundreds of thousands of people from universities, hospitals, biobanks.<br><br>GWAS data for 731 immune cells with different immune phenotypes from a sequence of 3757 individual European data from Sardinia, Italy.
11	**Main results**				
		a)	Report the associations between genetic variant and exposure, and between genetic variant and outcome, preferably on an interpretable scale	-	-
		b)	Report MR estimates of the relationship between exposure and outcome, and the measures of uncertainty from the MR analysis, on an interpretable scale, such as odds ratio or relative risk per SD difference	05	CD3 on CD39+ activated Treg was the only immune cell type that had positive causality for all three different airway allergic diseases (IVWAR = 0.001, IVWCRS = 0.043, IVWAS = 0.027) and was protective in all three diseases (ORAR = 0.940, ORAS = 0.967, ORCRS = 0.976) without heterogeneity or horizontal pleiotropy.
		c)	If relevant, consider translating estimates of relative risk into absolute risk for a meaningful time period	-	-

		d)	Consider plots to visualize results (e.g. forest plot, scatterplot of associations between genetic variants and outcome versus between genetic variants and exposure)	05	The final screen identified 38 immunophenotypes of immune cells (Supplementary Table S1), 12 different phenotypes of immune cells as risk factors for AR; Immune cells of 26 different phenotypes are protective against AR.<br>Fig.2-5
12	**Assessment of assumptions**				
		a)	Report the assessment of the validity of the assumptions	05	A total of 47 different cellular immunophenotypes significantly associated with AS were identified, among which 10 were removed due to significant heterogeneity.
		b)	Report any additional statistics (e.g., assessments of heterogeneity across genetic variants, such as I^2, Q statistic or E-value)	05	A total of 47 different cellular immunophenotypes significantly associated with AS were identified, and 10 were removed with significant heterogeneity.<br>Supplementary.1-3
13	**Sensitivity analyses and additional analyses**				
		a)	Report any sensitivity analyses to assess the robustness of the main results to violations of the assumptions	-	Supplementary Table 1-3
		b)	Report results from other sensitivity analyses or additional analyses	05	All IVs included in the final analysis had F-values greater than 10 and all results were tested for stability by funnel plots and leave-one-out tests.
		c)	Report any assessment of direction of causal relationship (e.g., bidirectional MR)	05	Reverse Mendelian results suggesting bidirectional causation of FSC-A on CD8br, CD4 on secreting Treg were eliminated
		d)	When relevant, report and compare with estimates from non-MR analyses	-	-
		e)	Consider additional plots to visualize results (e.g., leave-one-out analyses)	-	Fig 2-5
	**DISCUSSION**				
14	**Key results**		Summarize key results with reference to study objectives	07	Our study found that CD3 on CD39+ activated Tregs (CD4+ CD39+ CD25+++ CD45RA- CD127lo Treg) played a protective role in three types of AAD.

15	**Limitations**	Discuss limitations of the study, taking into account the validity of the IV assumptions, other sources of potential bias, and imprecision. Discuss both direction and magnitude of any potential bias and any efforts to address them	10	However, some limitations of our study exist, firstly, we appropriately expanded the inclusion when immune cells were used as exposure (p<1e-5), which may somewhat contribute to biased results and false positives, secondly, all our data sources are based on European populations and extrapolation to other races should be construed with caution, and lastly, because of the lack of personal information we were unable to stratify the population analysis any further. Additionally, it is regrettable that there is still a lack of expression Quantitative Trait Loci (eQTL) data for different phenotype immune cells in publicly available databases, which to some extent limits our ability to better understand and explore the role of different phenotype immune cells in AAD. Therefore, we will continue to focus on and explore the eQTL data of different phenotype immune cells in our future research to improve our study results. We believe that with the continuous improvement of public databases and the efforts of more researchers, the role of different phenotype immune cells in AAD will be better understood in the future, and new biomarkers and therapeutic targets will be discovered.
16	**Interpretation**			
	a)	Meaning: Give a cautious overall interpretation of results in the context of their limitations and in comparison with other studies	08-09	Our study found that CD3 on CD39+ activated Tregs (CD4+ CD39+ CD25+++ CD45RA- CD127lo Treg) played a protective role in three types of AAD. CD39 has been identified as an E-NTPD enzyme, predominantly expressed on the surface of Treg cells......new AAD treatment targets based on CD3 and CD39 in the future.<br><br>Although our findings suggest that CD28 on CD39+ secreting Treg may be a risk factor for AR, the absence of literature directly linking this specific phenotype with AR, together with the potential risk of false positives due to the selection of analysis thresholds in our study, necessitates further confirmation through clinical and cellular experimentation.
	b)	Mechanism: Discuss underlying biological mechanisms that could drive a potential causal relationship between the investigated exposure and the outcome, and whether the gene-environment equivalence assumption is reasonable. Use causal language	08	CD3+ is a phenotype shared by T cells, which can promote the activation and differentiation of Tregs, and enhance their immunosuppressive function.

			carefully, clarifying that IV estimates may provide causal effects only under certain assumptions		During the activation process of T cells, the CD3-TCR complex must first bind to antigenic peptide fragments presented by MHC on the surface of other antigen-presenting cells, subsequently activating the proliferation and inhibitory functions of T cells. Once T cells are activated, to limit the intensity of T cell activation, cells achieve the recycling and degradation of the CD-TCR complex through endosomal recycling.
		c)	Clinical relevance: Discuss whether the results have clinical or public policy relevance, and to what extent they inform effect sizes of possible interventions	08	In summary, our research results further emphasize the importance of CD3 on CD39+ activated Tregs in the development of airway allergic diseases from a causal perspective, and provide new insights and a basis for further exploration of new AAD treatment targets based on CD3 and CD39 in the future.
17	**Generalizability**		Discuss the generalizability of the study results (a) to other populations, (b) across other exposure periods/timings, and (c) across other levels of exposure	10	all our data sources are based on European populations and extrapolation to other races should be construed with caution
	**OTHER INFORMATION**				
18	**Funding**		Describe sources of funding and the role of funders in the present study and, if applicable, sources of funding for the databases and original study or studies on which the present study is based	10	The authors received research grants from The National Natural Science Foundation of China(81970865) and Four "Batches" Innovation Project of Invigorating Medical through Science and Technology of Shanxi Province(2022XM06) and Research Project Supported by Shanxi Scholarship Council of China (2021-172), The funder had no role in the study design, data collection and analysis, decision to publish, or manuscript preparation.
19	**Data and data sharing**		Provide the data used to perform all analyses or report where and how the data can be accessed, and reference these sources in the article. Provide the statistical code needed to reproduce the results in the article, or report whether the code is publicly accessible and if so, where	03	GWAS statistics for each immune cell population are available from the GWAS Catalog (GCST0001391-GCST0002121).
20	**Conflicts of Interest**		All authors should declare all potential conflicts of interest	11	The authors declare that the research was conducted in the absence of any commercial or financial relationships that could be construed as a potential conflict of interest.

This checklist is copyrighted by the Equator Network under the Creative Commons Attribution 3.0 Unported (CC BY 3.0) license.

1. Skrivankova VW, Richmond RC, Woolf BAR, Yarmolinsky J, Davies NM, Swanson SA, et al. Strengthening the Reporting of Observational Studies in Epidemiology using Mendelian Randomization (STROBE-MR) Statement. JAMA. 2021;under review.

2. Skrivankova VW, Richmond RC, Woolf BAR, Davies NM, Swanson SA, VanderWeele TJ, et al. Strengthening the Reporting of Observational Studies in Epidemiology using Mendelian Randomisation (STROBE-MR): Explanation and Elaboration. BMJ. 2021;375:n2233.
